# The New York Institute of Stomatology

**Published:** 1896-07

**Authors:** 

**Affiliations:** The New York Institute of Stomatology


### THE NEW YORK INSTITUTE OF STOMATOLOGY.

A meeting of the Institute was held on Tuesday evening, May 5, at the residence of Dr. Benjamin Lord, 34 West Twenty-eighth Street, the president, Dr. Lord, in the chair. The minutes of the last meeting were read and approved.

The president introduced Dr. Dawbarn, who brought with him for exhibition before the Institute two patients. One was a young girl with a non-malignant type of epulis springing from the socket of the upper right first molar. It had been first noticed nearly two years before, and had not grown much since then. It was at this time not larger than a pea. Of late it had seemed to be the starting-point of attacks of facial neuralgia upon that side.

The other patient was a young woman who developed the sarcomatous type of epulis—as proved by microscopical examination—springing from the sockets of both bicuspids upon her upper right side, and being of nearly a year's duration when Dr. Dawbarn first saw her. He operated by removing all the teeth on that side save the central incisor, and then chiselling away the entire alveolar process for a corresponding distance. Healing was prompt and complete, but after two months there was some recurrence at two points. For this reason Dr. Dawbarn entirely excised both of her external carotid arteries, making two operations for the two sides, as it is a long dissection. The object was to starve out the growth by shutting off its main blood-supply. This succeeded, as both places of recurrence gradually disappeared, leaving sound healing again. Whether there may ultimately be again a return only time can show. She will be watched closely, and in that case the entire right upper jaw must be excised. Because the resulting deformity from this would ruin any chance of marriage, Dr. Dawbarn thought the attempt to save the jaw justifiable.

The president thanked Dr. Dawbarn for so kindly exhibiting the patients, and commended him for his skilful treatment of them, and then called upon Dr. W. St. George Elliott, the chairman of the Committee upon Recent Dental Literature for the report of the committee, which was read by Dr. Elliott.

### REPORT ON RECENT DENTAL LITERATURE.

Your committee beg to report that they have given the matter of a report on recent dental literature a good deal of thought, and while believing that such report might be made of importance, yet there are many difficulties in the way. Your committee, like most committees, cannot get together often, and were they to do so, could not work harmoniously, the difficulty being that no two members can have the same views as to the importance or non-importance of certain articles. In this first report we beg to offer some suggestions. First, that only those be appointed on this committee who are willing to bestow the necessary time and labor; second, that we report but once a year.

We think there is no subject now before the profession so important and so interesting as that of cataphoresis, and there are several articles in the journals worthy of study. We know that cataphoresis is but the forcing into the system of various medicines, through the agency of the electric current, for various purposes; that a pressure of a maximum of, say, twenty volts is necessary with a current of less than an ampère; that an essential feature is that the pressure must be turned on, increased, and decreased gradually, or pain will result. With these essentials before us, we can more clearly look into the various statements made. Our friend Dr. Morton contributes to the January *Dental Cosmos* a paper on the subject, and brings forward the combination of guaiacol with cocaine, the former containing eight per cent. of the latter; the combination of these two sedatives are supposed to act much more promptly than when given alone. This idea does not seem to be sustained by any experiments or proofs. Drs. Morton and Gillett both bring forward the Wheeler volt-selector, an apparatus for turning on the current slowly; it is misnamed a volt-selector, and is merely a suitable reostat. There are several ways of obtaining the current. First, the street current, of one hundred and fifteen volts, passed through a suitable resistance, as the Wheeler volt-selector, to the patient; second, the use of a primary battery with resistance; and, third, the combination, or, more properly, the street current passed through a storage battery. By the means just alluded to—the street current passed through resistance, and thus to the patient—we meet with the serious objection that the street voltage varies as high as ten per cent. in pressure, and one per cent. sudden increase or decrease of pressure produces pain. In the second process, a primary battery is used, with resistance. This plan has much to commend it, for the apparatus is portable and efficient, the only objection being the cost. A good dry-cell battery, with milliampère meter and switch, costs some fifty dollars, and, with full use, will not last over a year, when all the cells must be replaced. In the third process, the storage cells (ten), of two volts each, and costing five dollars and fifty cents each, are kept saturated by connection with the street current, while switches, milliampère meter, and perhaps voltmeter, with resistance, form the necessary apparatus.

Drs. Morton and Gillett use the first form of current,—street, Wheeler volt-selector, etc.,—while Drs. J. Morgan Howe, Van Woert, and others the primary battery. Dr. Brown, of Montreal, uses the street current through storage cells, and Dr. Rollins uses thirty-volt candle lamps as resistance, together with a rotary resistance, probably the familiar German-silver wire resistance-box, taking his current from the street. There is sufficient evidence now before the profession to prove that there is something of value to us as dentists in cataphoresis, but it is not an unmixed blessing, and does not prove of value in every case. The present objection to the apparatus is the time required, averaging, say, fifteen minutes. But experimental efforts should reduce this. One minute is long enough; indeed, we see no reason why it may not be made so rapid that we can carry the cocaine into the dentine by the use of our excavators while in the act of excavating. The objection at present to this, in addition to the time, is the pain from making and breaking circuit when the instrument is applied and removed. This might be obviated by using a large current with low voltage; say, ten ampères and one volt.

In the January *Dental Cosmos* there is an interesting article by Dr. W. E. Walker, of Mississippi, calling attention to an unnoticed anatomical fact,—*i.e.*, the downward slope of the roof of the glenoid fossa. It is for this reason, the doctor states, that a set of artificial teeth articulate perfectly in the articulator, but failed to do so when placed in the mouth. Natural lateral motion in the lower jaw must be accompanied by a downward one as well. Dr. Walker states that this angle varies in different individuals from twenty-two to forty-five degrees, and in his own articulator the angle is made adjustable.

Dr. Black, of Illinois, has an excellent article on the influence of oxidation in alloys to produce contraction. His experiments prove his statement, but the older members of our profession remember when we were all told to work away the oxide with alcohol, and how, years afterwards, we were told not to work them. Here we have a text upon which we might expatiate all night. There has been a tendency in the profession, unfortunately, to follow any one who makes a statement, without regard to proofs. Thanks to Dr. Miller, Dr. Black, and others, we are now more incredulous, and want at least some evidence to support assertions.

In the *Journal of the British Dental Association*, Rutherford, of London, advertises a new crown, all porcelain, the only novelty being that the hole in the crown is oval, and a flattened pin, cemented in, is used.

Dr. Strang, of Bridgeport, Conn., recommends the combination of oxyphosphate and amalgam. First alloy is mixed with mercury. Then phosphate powder added, and finally liquid phosphoric acid.

In an editorial in the *Items* there is a good deal said of injury to the teeth by the tooth-brush, and this statement is made: "It is a shame to see the destruction of so many sets of nice teeth from this cause." Perhaps Dr. Welch's opportunities for investigation may have been remarkably good; at all events, the chairman of this committee, in his twenty-six years of dental practice, has yet to see the first set of teeth ruined in this way, while an innumerable number have been vastly injured by the lack of thorough cleansing. Your committee beg to state their belief that it is not possible to produce a true case of erosion by the tooth-brush. With the great majority of these cases there is a sharp edge, and it is impossible for this edge to be produced with the brush; on the contrary, brushing the teeth would soon destroy the sharp edge. Patients should always be instructed to brush from the gums, as being more efficacious than across the teeth.

Dr. Sudduth, of Minneapolis, has a good article in the February *Dental Cosmos* on pyorrhœa, mechanical removal of irritants, pyrozone thirty per cent. and cocaine in pockets, and in extreme cases zinc chloride, with other medicaments.

Dr. Driscoll, of Manatee, Fla., describes his mode of using oxyphosphate and amalgam. First introduce the mixed phosphate into the cavity, and, before setting, press into the cement a button of alloy. There are some objections to this plan, unless great care is used to prevent the cement getting to the edges.

Dr. A. H. Brockway spoke favorably of the report as read by Dr. Elliott, and said it had always been a favorite idea of his that the interests of a dental society could be furthered by occasional reference to and discussion of papers already published in the journals. For that reason he expected good to result to the society from the work being done by Dr. Elliott's committee.

The Committee on Operative Dentistry, Dr. E. A. Bogue, chairman, stated that the report of that committee would not be read for lack of time, but would be presented at a later meeting still further amplified. An extract from Dr. McNaughton's portion of the report was read, because it gave the formula for his obtundent. This obtundent is composed of equal parts cocaine and thymol made by heating the thymol in a test-tube until it is melted and dissolving the cocaine in it. For sensitive dentine a piece of asbestos paper is saturated with this and placed in the cavity over as broad a surface as possible, and this is covered with zinc phosphate for several days.

The remainder of the report was stated to consist of experiments with the new volt-selector, so extensively advertised at the present moment as serving to obtund the sensibility of teeth by cataphoresis.

The committee found that from fifteen to forty minutes were required to secure immunity from pain with this particular instrument, and that the process was very disagreeable and not free from dangers of various kinds, not the least of which was the danger of so thoroughly displeasing the patient as to permanently offend him. The results of a few experiments on bleaching teeth are promised for a later date.

*The President.*—There is now an opportunity for any who wish to speak upon this subject of cataphoresis. We would be glad to hear from Dr. Allan, of Newburgh.

*Dr. C. F. Allan.*—I would like to say that the experience of Dr. Bogue in the use of cataphoresis has not been mine in the one extreme case in which I have tried it. Some weeks ago, Dr. Van Woert was kind enough to bring his apparatus to Newburgh, and I had a patient on hand, a young lad of thirteen or fourteen years, and for whom I had found it difficult to work, who was suffering from a deep crown cavity in an upper second molar, and in whose mouth our ordinary remedies for obtunding pain were quite useless. The boy wanted to do his part, tried hard always to bear the necessary pain, and simply found it impossible. He was evidently anxious and fearful of this new method, and the use of electricity had to him the meaning of something strange and fearful.

One had to be very gradual in the turning on of the current, and it was thought best to keep it on an unusually long time, three-quarters of an hour, but at the end of that time there was no more pain than if I had been working on a block of wood, and the boy was delighted. I could use the bur with the utmost freedom, and the turning up of the leathery decay and the completion of the filling was without any apparent resuscitation of feeling.

I have seen the patient several times since, and there have been no unpleasant after-effects, such as spoken of by Dr. Bogue. A twenty-five-per-cent. solution of cocaine was used.

I would say that with the lack of fear, the result of his practical experience of this method of treatment, this same patient would require probably not half the time in a future case to accomplish the same result.

*Dr. G. S. Allan.*—The Executive Committee has received a communication from Dr. Jack, of Philadelphia, upon this subject, which, with the president's permission, I will ask the secretary to read.

*Dr. Louis Jack.*—As Dr. Allan will inform you, I have been using acceptably the apparatus No. 11 of the Chloride of Silver Dry-Cell Battery Company, of Baltimore. This was chosen because it has been efficient for medical purposes, and further, that it had attached to it the Willms controller, which gives the most gradual gradation of the resistance. This commences with a resistance of ninety thousand ohms, which is reduced by one hundred and twelve contacts, and therefore these furnish a very slow gradation.

The well-known fact that the chloride of silver cell is extremely constant, and that it has a ratio of voltage to ampèrage which is acceptable to the human tissues, were further reasons to lead me to adopt this source of current.

The voltage of each cell is one and the ampèrage between one-fifth and one-fourth of one ampère.

I have used eighteen- and twenty-four-per-cent. solution of cocaine hydrochloride and of the citrate, the latter appearing the most efficient. The citrate at present is procurable of Merck & Co., of New York.

A most important consideration concerning the application, as it appears to me, is that the force of the current be not great. I have found ten to twenty volts efficient, and that this degree of force has been sufficient generally to produce the effect desired. For children I use ten or fifteen cells; for adults, fifteen or twenty cells. It is evident that if the voltage be high the necessary consequence must be the evolution of heat in the tooth, which may have some influence upon the after condition of the tooth. Thus far no secondary symptoms have appeared in my practice.

It may be interesting to give the history of four clinical cases, each of which were extremely sensitive to the lightest touch.

Case I.—Buccal aspect of lower bicuspid cavity broad but not deep; applied cocaine hydrochloride sixteen per cent., with the addition of two drops of carbolic acid. Selected twenty cells; controller-switch carried to 180°, or fifty-second pin. Time, fifteen minutes; relief nearly complete, some pain being indicated at the extreme lateral margins when grooving.

Case II.—Small cavity in proximate surface of central, which had been cleansed, but the sensibility forbid completion. First applied cocaine hydrochloride eighteen per cent. for twelve minutes, which did not completely remove sensibility; then applied the citrate, same percentage, for a less period, with absolute relief. There was no record of the voltage.

Case III.—Lower bicuspid; occlusal surface denuded of enamel, apparently dense; cocaine citrate sixteen per cent., twenty cells, selected; time, twelve minutes. Considerable cutting was made to form cavity when some acuteness appeared; reapplied same solution with twenty-five cells for same period, when the excavation was completed without pain.

Case IV.—Lower molar buccal surface at cervix, including part of mesial aspect; cocaine hydrochloride twenty-four per cent.; time, twelve minutes of twenty cells, and continued eleven minutes more with twenty-five cells. Complete excavation and retention made without pain.

The two latter cases were typically severe ones, which required nearly twice the usual time. Dr. Allan, having been present, will be able to describe the results.

*The President.*—I now have the pleasure of introducing one of our members, Dr. Samuel A. Hopkins, of Boston, who will read a short paper on cocaine.

(For Dr. Hopkins's paper, see page 431.)

DISCUSSION.

Dr. Dawbarn was called upon by the president, and stated that while in the main he cordially agreed with the thoughts as expressed by Dr. Hopkins, yet he must differ regarding the view expressed as against the use of cocaine in surgery because of its danger. There is no drug of real value to us which is not a two-edged sword, and capable, if misused, of doing harm. This argument might with equal force be brought against ether anæsthesia.

He thought the use of cocaine in surgery was on the increase, and, used carefully and with certain precautions, it was safe. Personally, he had had no case of poisoning from its use, almost daily, for a period of several years. His colleague, Dr. Wyeth, at the Polyclinic School and Hospital, uses it even more than he, and at times for major operations, such as amputation at the shoulder, and ligations of the subclavian artery, and regards a four-per-cent. solution as best for injection.

Dr. Dawbarn thought it a safe rule not to have more than one grain absorbed, and always precedes its injection by giving the patient a large drink of whiskey, or else a hypodermic injection of glonoin—also called trinitrin, and which comes in tablet-triturates of $\frac{1}{100}$ grain each—is given together with the cocaine. Either alcohol or glonoin will dilate blood-vessels, and thus balance the tendency of cocaine to contract them unduly and thus to cause anæmia of the brain.

Should symptoms of faintness arise, the patient should be placed flat, and given by the mouth two drachms of aromatic spirits of ammonia, in water, or more whiskey.

Whether, as Dr. Hopkins believes, there is a greater liability to unpleasant symptoms following the use of cocaine about the head than elsewhere he could not say.

Regarding the reference to Dr. Schleich, of Berlin, Dr. Dawbarn believed that his method of anæsthesia, produced by injection of extremely weak and almost infinitesimal doses of cocaine, is mainly a repetition of the work of Dr. Halsted, professor of surgery at the Johns Hopkins Hospital in Baltimore. Some twelve years ago he had seen Dr. Halsted remove a superficial tumor painlessly by the use of plain water injected into the skin surrounding the growth. Indeed, even before that time Bartholow, in his "Materia Medica," had, in the chapter upon "Aquapuncture," pointed out that water is anæsthetic to a considerable extent, and advised its use injected in contact with the sciatic nerve in obstinate sciatica.

Dr. Dawbarn remarked that he was greatly interested in electric cataphoresis, which he had used for opening felons, and similar conditions, a good many years ago. He believed that Dr. Frederick Peterson, who devised this term, credited him with being the first surgeon to do this. But it was a slow method, and he had subsequently employed cocaine, ethyl chloride, or other means, by preference. In dentistry, however, he could see the possibility of a brilliant future for electrolysis.

In conclusion, Dr. Dawbarn said that he should like to have the Institute take occasion to try one or all of the following anæsthetic drugs, hoping that among them may be found one superior to cocaine for dental work. These are—

"*Tropsin*" (first described by Schweigger, of Berlin, see *New York Medical Record*, October 8, 1892). A three-per-cent. solution produces eye-anæsthesia more quickly than does the same strength of cocaine. It is obtained from the small-leaved cocoa-plant of Java. Chemically, tropsin is closely related to atropine, and is really benzoyl tropeine.

*Brucine*, obtained from nux vomica seeds; save that it is anæsthetic, it has the same action as strychnine, but is considerably less powerful than the latter.

*Strophanthin*, obtained from the seeds of strophanthus hispidus, which is the kombé arrow-poison of tropical Africa. Used internally, in proper dosage, strophanthus is a heart-tonic.

*Erythrophlœine*, from sassy bark (E. guineense), which is from Africa. Dr. Carl Koller, the discoverer of the properties of cocaine, has recommended it recently. (See *New York Medical Journal*, March 30, 1895.)

*The President.*—We will now have the pleasure of hearing from Dr. F. T. Van Woert, of Brooklyn, who has been kind enough to join us in this evening devoted to the consideration of "local anæsthesia," and who will not only relate his experience with cataphoresis, but will also exhibit the apparatus he uses, some portions of which, I understand, he devised.

*Dr. Van Woert.*—I want, in beginning, to correct the impression left by the remarks made by Dr. Bogue. From what he has said it might be inferred that the Wheeler apparatus for cataphoric medication is absolutely worthless. On the contrary, I know from practical experience that perfect results can be obtained by its use, and my only objection to it is that it is not quite as practical and handy for dental purposes as we could wish. The time consumed in its use is considerably longer than need be, and there is no positive accuracy in its registration.

The instrument which I have to present to you this evening has a maximum voltage of thirty-one and a half, or one and a half volts to a cell, there being twenty-one cells in the outfit. I am not in favor of the use of the street current for this purpose, and I find it is unnecessary, because the voltage required is so small, averaging from five to nine volts only, that the batteries are very much preferable and certainly are safer. The general construction of the instrument is such that the amount of pressure and the volume of current used can be divided and read without a technical knowledge of electricity; and to my mind the use of the milliampère meter, as well as the graduation of the current-controller, is absolutely necessary to the successful use of electricity for cataphoric medication. For instance, a patient presents himself, and we find upon application of the current a resistance of one-tenth of a milliampère in volume against less than a volt in pressure. There can be no doubt that the resistance in this case is so high that it will consume a great deal more time than one where the milliampère meter would register three-fifths of a milliampère against three to five volts in pressure; from which you can readily see the necessity of being able to know the amount of pressure and the volume of current passing through the tooth. After my few months experience I feel that I am able to tell almost to a moment how long it is going to take to anæsthetize the part, simply by comparing the volume against the pressure of current consumed.

One of the most important factors in cataphoric medication is the proper conductibility of the medicament used. Professor Morton has recommended the use of guaiacol in connection with cocaine. Guaiacol I find, like all of the oils or fats, is an absolute non-conductor, and unless sulphuric acid or some chemical having high conductive properties is added, it would be of little use. I find in my practice the best results are obtained from an aqueous solution,—thirty per cent.,—or a saturated solution, of cocaine, with a very slight trace of chloride of sodium. The conductibility of plain water with a pressure of thirty-one and a half volts is just three-tenths of a milliampère to the half drachm,—that is to say, when thirty-one and a half volts are running through half a drachm of water the milliampère meter will show three-tenths of a milliampère, and a milliampère is a thousandth part of an ampère. Thus you can see that water is a very poor conductor.

It has been stated that bleaching of teeth is very much facilitated by the use of the electric current, and it is recommended that a twenty-five-per-cent. solution of pyrozone be used. Upon careful experiment you will find that pyrozone solutions, either five per cent. or twenty-five per cent., are absolute non-conductors, and the addition of the electric fluid does not hasten or facilitate the operation in the least.

One of the most difficult obstacles to overcome is found in the application of the current to the tooth. If it is to be necessary for a busy man to stand with an electrode in his hand from five to thirty minutes, that will very soon place cataphoresis on the top shelf in the back office. To overcome this difficulty I have devised several electrodes, made from German silver spring wire, which can be snapped into place and allowed to hang without the aid of an assistant, and the operator, after adjusting them, may proceed with other obligations of the day, leaving to the office-boy, girl, or assistant such slight additions of current as the case may require. Personally, I arrange my appointments so that patients with sensitive teeth that need this anæsthetizing process shall present themselves at least one hour before I am ready for the operation. I place them in an ordinary chair, give them something interesting to read or look at, and after applying the dam and making the proper connections with the apparatus, I go on with other appointments, leaving the patient in charge of my young lady assistant during any time that I may be occupied prior to their taking the operating-chair; then, when I am ready for the operation, the teeth are absolutely insensible to the feeling, and I am able to excavate and fill without the least demonstration on the part of the patient, from which you can understand that the time consumed in the operation will be at least one-third, if not one-half, less than if I were to attempt it without anæsthetizing the part. The only objection raised against cataphoresis is the time that it consumes, and if the time saved in the possibility of rapid operations following the cataphoric application be placed against the time consumed in its use, you will find that, by deducting the former from the latter, the time consumed in all will be very little more than it would be if electricity were not used, to say nothing of the great relief from nervous strain to operator and patient. I have come to look upon my Saturday mornings with as much comfort and freedom as any other day in the week since the introduction of this method, while prior to that time Saturday was a skeleton in my closet, and I have felt for many years that if I could ever acquire a competency, I would abandon the treatment and filling of children's teeth.

The anæsthesia of sensitive dentine and the extirpation of living pulps form a small part only of the uses to which this apparatus may be put. In the treatment of pyorrhœa alveolaris I make an application of cocaine to the soft and hard tissues for from five to ten minutes, after which I am able to cut and scrape to my heart's content without interference because of pain to the patient. Again if a case of acute pericementitis comes into the office, I immediately apply a half strength tincture of iodine by means of one of the little cup electrodes which you see here, giving about twelve or fifteen volts from three to five minutes, and if you try that you will find that in the majority of cases the pain will cease in a very few moments and the trouble entirely disappear. There are other cases in which cataphoresis may be usefully employed, as in the introduction of sterilizing or germicidal agents into the roots and tubuli of the teeth, the deposition of a compound of zinc and copper, known as electrolysis, for the destroying of sacs where pus is forming, better known as alveolar abscesses, and in suppurative processes, such as pyorrhœa alveolaris. In short, the field seems to be unlimited, and I can hardly realize where it will end.

I shall be very glad to carefully go over the details of this matter, and the instruments presented, with any of the individual members after the meeting, and I may say that it is utterly impossible to give a description extemporaneously and without preparation which every one would understand.

I feel sure that cataphoresis has come to stay in dentistry. The man who practises without it is going to be considered a back number within a very few months, and he will find his patients drifting around the corner to Dr. Jones, his neighbor, for the relief which humanity has been seeking ever since science was known.

The president personally thanked all who had taken part in the meeting, and congratulated the Institute upon the large attendance and the great interest shown.

The Institute passed a vote of thanks to Drs. Dawbarn, Hopkins, and Van Woert for their valuable contributions.

Adjourned.

S. E. Davenport, D.D.S., M.D.S.,
*Editor The New York Institute of Stomatology.*

